# Mitigating the Risk of Drug Interactions in Cancer Patients Taking Oral Anticancer Agents: The Role of a Multidisciplinary Team-Based Medication Reconciliation

**DOI:** 10.7759/cureus.35324

**Published:** 2023-02-22

**Authors:** Jorge Rodrigues, Paula Marques, Catarina Gomes, Catarina Portela

**Affiliations:** 1 Medical Oncology Department, Hospital de Braga, Braga, PRT; 2 Hospital Pharmacy Department, Hospital de Braga, Braga, PRT

**Keywords:** hospital pharmacy, hospital and clinical pharmacy, medication reconciliation, drug utilization review, oncology, drug-drug-interactions

## Abstract

Purpose: Polypharmacy in cancer patients is a recognized issue and should be an integral part of comprehensive patient assessment and management. Despite this, a systematic review of concomitant drugs or a search for potential drug-drug interactions (DDIs) is not always performed. Here, we present the results of a medication reconciliation model performed by a multidisciplinary team to identify clinically meaningful potential DDIs (defined by the presence of DDI of major severity or contraindication) in cancer patients undergoing oral antineoplastic drugs.

Methods: From June to December 2022, we performed a non-interventional, prospective, cross-sectional, single-center study of adult cancer patients, initiating or undergoing treatment with oral antineoplastic drugs, referred by their oncologists for therapeutic review regarding potential DDIs. DDIs were assessed by a multidisciplinary team of hospital pharmacists and medical oncologists, through research in three different drug databases as well as in the summary of product characteristics. A report detailing all potential DDIs was created for each request and provided to the patient's medical oncologist for further examination.

Results: Overall, 142 patients’ medications were reviewed. Regardless of the severity or clinical importance, 70.4% of patients had at least one potential DDI. We found 184 combinations of oral anticancer and regular therapy agents with potential DDIs, 55 of whom were considered of major severity by at least one DDI database. As expected, the number of potential DDIs increased with the number of active substances in regular therapy (*p < *0.001), but we did not find an increased relation between age and the total number of potential DDIs (*p = *0.109). Thirty-nine (27.5%) patients had at least one clinically meaningful DDI identified. After adjustment through multivariable logistic regression, only the female sex (odds ratio (OR) 3.01, *p* = 0.029), the number of active comorbidities (OR 0.60, *p* = 0.029), and the presence of proton pump inhibitors in chronic medication (OR 2.99, *p* = 0.033) remained as predictors of potential meaningful DDI.

Conclusion: Although drug interactions are a concern in oncology, a systematic DDI review is rarely conducted in medical oncology consultations. The availability of a medication reconciliation service, carried out by a multidisciplinary team with dedicated time for this task, is an added value for safety enhancement in cancer patients.

## Introduction

In the Western world, it is estimated that around 30% of adults over 65 years of age have five or more medications in their regular therapy [1]. Although polypharmacy may be necessary for many patients, it increases the risk of potentially avoidable drug-drug interactions (DDIs), sometimes associated with major and potentially fatal adverse effects [2-4]. Furthermore, it is well known that the number of drugs in co-medication (counted as the number of pharmacologically active components) is the single best predictor for the risk of iatrogenesis [5,6].

In oncology, DDI acquires additional relevance in clinical practice. Cancer patients often require multiple medications not only to manage their disease but also for treatment-related side effects. Likewise, most anticancer agents have a narrow therapeutic index and a more exuberant toxicity profile. This increases the risk of DDI, which can occur when two or more drugs interact in a way that changes the effectiveness or toxicity of one or more of the medicines. These interactions can lead to increased side effects, reduced treatment effectiveness, or even harm to the patient [7-9].

Polypharmacy in patients with cancer is a recognized issue worldwide and should be an integral part of comprehensive cancer patient assessment and management [10]. Despite this, we know that a systematic review of concomitant drugs or a search for potential DDI is not always performed [10,11]. This may happen for various reasons, such as a shortage of time in the medical consultation to review therapy, incomplete information provided by patients and families regarding concomitant drugs, including over-the-counter medications and dietary supplements, lack of a medication reconciliation carried out by a pharmacist, lack of access to databases for DDI search (not always with consensual information), among others. Furthermore, in recent years, we have seen an increase in the number of oral anticancer therapeutic options, which offer greater convenience to patients in terms of administration flexibility, comfort, and, in many cases, an increase in the quality of life. However, it is also known that patients undergoing oral antineoplastic therapies maintain these treatments for a longer period and are therefore at a greater risk of potential DDI [9,12]. Indeed, new challenges have emerged with the use of oral anticancer agents (e.g., tyrosine kinase inhibitors, cyclin-dependent kinase inhibitors, and poly(adenosine diphosphate-ribose) polymerase inhibitors, among others). These drugs are highly susceptible to DDI, which may include, among other examples, an impact on pharmacokinetics (absorption, distribution, metabolism by cytochrome P450 enzymes, clearance) and additional pharmacodynamic effects (additive, synergistic or antagonistic effects) such as the combination of heart rate-corrected QT interval (QTc)-prolonging drugs [9,13].

There are several DDI platforms and databases that are widely used in oncology and other medical fields. Some of the most used are Micromedex [14], Lexicomp [15], Drug Interaction Checker (Drugs.com) [16], RadBoudUMC Database [17], Medscape Drug Interaction Checker [18], and Epocrates (access not available in European countries) [19]. Although some of these databases are open-access, others may require a paid subscription. Furthermore, DDIs are complex, and a database search may not capture all potential interactions. It is also possible that different DDI platforms or databases may have different information for the same anticancer drug [20,21]. Therefore, to have a more complete understanding of a potential DDI, healthcare providers need to consult multiple DDI platforms or databases. We would also like to stress the importance of clinical judgment when assessing potential DDI in every individual patient.

The shortage of time in medical consultation to make a complete DDI review reinforces the need to have a multidisciplinary team dedicated to this task, providing a medication reconciliation service.

The primary endpoints of this study were as follows: (a) identification of clinically meaningful potential DDI in cancer patients undergoing oral antineoplastic drugs and (b) characterization of the frequency of potential DDI in our population. Secondary endpoints were as follows: (a) validation of a medication reconciliation model implemented by a multidisciplinary team of hospital pharmacists and medical oncologists to identify potential DDIs early and enhance patient safety and (b) evaluation of baseline demographic and clinical patient characteristics on DDI.

## Materials and methods

We performed a non-interventional, prospective, cross-sectional, single-center study from June to December 2022. Cancer patients aged 18 or older, initiating or undergoing treatment with oral antineoplastic drugs, followed at our center (Medical Oncology Department of Hospital de Braga, Portugal) were referred to our project by the attending medical oncologist for therapeutic review regarding potential DDI. After obtaining informed consent from the patient following a thorough explanation of the project, a review of all current medications was conducted. Potential DDIs were assessed through research in three different drug databases (Lexicomp Drug Interactions®, Drug Interaction Checker-Drugs.com, and RadBoudUMC Database-Cancer Drug Interactions) as well as in the summary of product characteristics (available at the EMA-European Medicine Agency website) [22]. This analysis was carried out by a multidisciplinary team consisting of hospital pharmacists and medical oncologists. A report detailing all potential DDIs was created for each request and provided to the patient's medical oncologist for further examination. 

Demographic and clinical patient data were collected. Categorical variables are presented as frequencies and percentages, and continuous variables as means and standard deviations (SD), or medians and interquartile range (IQR) for variables with skewed distributions. Normal distribution was checked using skewness, kurtosis analysis, and Shapiro-Wilk test. An independent sample *t*-test was used to compare independent continuous variables with normal distribution, and the Mann-Whitney *U* non-parametric test was employed for non-normal continuous variables. 

A regression curve estimation was performed between the dependent variable “number of potential DDIs” and the independent variable “number of active substances in regular therapy”. Also, we perform a logistic regression between the dependent variable "clinically significant potential DDI" and the different clinical and demographic variables from our patients. The Hosmer-Lemeshow test was used to check the goodness of fit.

For sample size assessment and statistical analysis, we estimated that 10% of cancer patients would have clinically significant potential DDI (defined by the presence of DDI of major severity or contraindication). Based on a type I error of 5% (*p *< 0.05) and an absolute error of 5%, we calculated the need to recruit at least 138 patients [23].

All *p*-values were two-sided at a significance level of 0.05. Statistical analyses were performed using IBM SPSS Statistics for Macintosh, Version 27.0 (Released 2020; IBM Corp; Armonk, New York, United States).

The local data protection office and hospital ethics committee reviewed and approved the project. 

## Results

A total of 142 patients were included in our study, 61 (43%) of whom were female, with a mean age of 64 (SD 11.4) years. Most of the patients had metastatic solid tumors (81.7%), with breast cancer (28.0%) and prostate cancer (30.1%) being the most represented. Clinical and comorbidity characterization and antineoplastic drugs reviewed are described in Table 1 and Table 2, respectively. We did not find a statistical difference in the number of active substances in regular therapy between both sexes (median 7.0 (IQR 4) in female patients versus 7.0 (IQR 7) active substances in male patients; *U* = 2316.5, *p* = 0.524). 

**Table 1:** Demographic and clinical characterization of the population. *Metachronous breast and ovarian cancer diagnosis in one patient. #Other hematological diseases: nodular sclerosis classical Hodgkin lymphoma (*n *= 1); b-cell chronic lymphocytic leukemia (*n *= 1); immune thrombocytopenic purpura (*n* = 1); essential thrombocythemia (*n *= 1). ECOG: Eastern Cooperative Oncology Group; IQR: interquartile range; NEC: neuroendocrine carcinoma; NET: neuroendocrine tumor; SD: standard deviation; PPIs: proton pump inhibitors.

	*N* = 142
Age, mean (SD)	64.0 (11.4)
≥65 years old, *n* (%)	77 (54.2)
Female, *n* (%)	61 (43.0)
ECOG Performance Status <2, *n* (%)	133 (93.7)
Cancer diagnosis	
Prostate cancer, *n* (%)	43 (30.1)
Breast cancer, *n* (%)*	40 (28.0)
Central nervous system tumors, *n* (%)	11 (7.7)
Renal cell cancer, *n* (%)	10 (7.0)
Colorectal cancer, *n* (%)	7 (4.9)
Hepatocarcinoma, *n* (%)	7 (4.9)
Ovarian cancer, *n* (%)*	6 (4.2)
Gastrointestinal stromal tumor, *n* (%)	5 (3.5)
Chronic myeloid leukemia, *n* (%)	4 (2.8)
Other hematologic malignancies, *n* (%)^#^	4 (2.8)
NEC or NET of gastrointestinal tract, *n* (%)	3 (2.1)
Lung cancer, *n* (%)	1 (0.7)
Carcinoma of unknown primary, *n* (%)	1 (0.7)
Cholangiocarcinoma, *n* (%)	1 (0.7)
Metastatic disease, *n* (%)	116 (81.7)
Presence of comorbidities, *n* (%)	129 (90.8)
Number of comorbidities, median (IQR)	2.0 (2.0)
Diabetes mellitus, *n* (%)	32 (22.5)
Arterial hypertension, *n* (%)	83 (58.5)
Ischemic heart disease, *n* (%)	5 (3.5)
Heart failure, *n* (%)	4 (2.8)
Peripheral vascular disease, *n* (%)	1 (0.7)
History of a cerebrovascular event, *n* (%)	6 (4.2)
Pulmonary thromboembolism, *n* (%)	3 (2.1)
Chronic obstructive pulmonary disease, *n* (%)	6 (4.2)
Chronic kidney disease, *n* (%)	1 (0.7)
Chronic liver disease, *n* (%)	8 (5.6)
Depression, *n* (%)	24 (16.9)
Other comorbidities, *n* (%)	101 (71.1)
Charlson Comorbidity Index Score, mean (SD)	8.1 (5.9)
Antineoplastic treatment indication, palliative, *n* (%)	130 (91.5)
Number active substances in chronic medication, median (IQR)	7 (4.8)
(Min - Max)	(1 - 18)
Number of patients with ≥ 5 active substances, *n* (%)	109 (76.9)
Number of patients with ≥ 10 active substances, *n* (%)	36 (25.4)
PPIs in chronic medication, *n* (%)	84 (59.2)
Patients with potential drug interactions, *n* (%)	100 (70.4)
Number of potential drug interactions per patient, median (IQR)	3 (2.0)
Patients with potential major drug interactions, *n* (%)	38 (26.8)
Patients with potential contraindication drug interactions, *n* (%)	1 (0.7)

**Table 2:** Antineoplastic drugs of cancer patients referred for DDI search. DDI: drug-drug interaction.

	*N* (%)
Abiraterone	29 (20.4)
Palbociclib + letrozole	12 (8.5)
Apalutamide	10 (7.0)
Ribociclib + letrozole	10 (7.0)
Temozolomide	10 (7.0)
Capecitabine	9 (6.3)
Imatinib	7 (4.9)
Sorafenib	6 (4.2)
Sunitinib	6 (4.2)
Enzalutamide	4 (2.8)
Axitinib	3 (2.1)
Olaparib	3 (2.1)
Ribociclib + fulvestrant	3 (2.1)
Cabozantinib	2 (1.4)
Everolimus	2 (1.4)
Lapatinib + exemestane	2 (1.4)
Niraparib	2 (1.4)
Palbociclib + fulvestrant	2 (1.4)
Tamoxifen	2 (1.4)
Trifluridine/tipiracil	2 (1.4)
Abemaciclib + fulvestrant	1 (0.7)
Abemaciclib + letrozole	1 (0.7)
Anastrazole	1 (0.7)
Azathioprine	1 (0.7)
Capecitabine + temozolomide	1 (0.7)
Dabrafenib + trametinib	1 (0.7)
Dasatinib	1 (0.7)
Everolimus + tamoxifen	1 (0.7)
Exemestane	1 (0.7)
Hydroxyurea	1 (0.7)
Lomustine	1 (0.7)
Osimertinib	1 (0.7)
Procarbazine	1 (0.7)
Regorafenib	1 (0.7)
Tucatinib + capecitabine	1 (0.7)
Venetoclax	1 (0.7)

**Figure 1:**
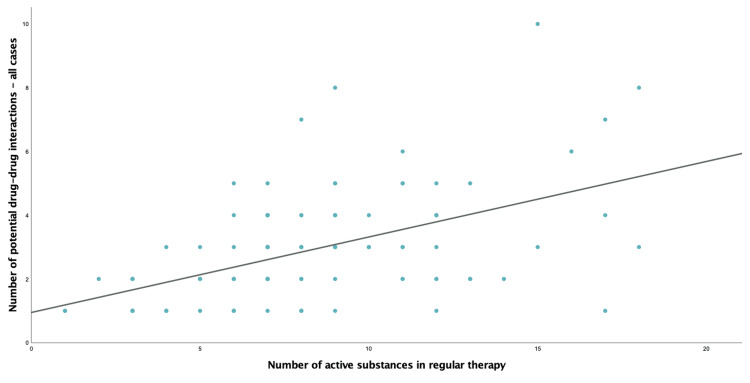
Relation between the number of potential DDIs and the number of active substances in regular therapy. Regression curve estimation (*R*^2^ = 0.264, *F*(1, 98) = 35.11, *p* < 0.001). DDI: drug-drug interaction.

In the present study, 70.4% of patients demonstrated at least one potential DDI, regardless of its severity or clinical significance. Of the total population, 184 combinations of oral anticancer and regular therapy agents were identified as the cause of 292 potential DDIs. Fifty-five combinations were considered of major severity by at least one DDI database. Consistent with previous data, the number of potential DDIs (all degrees of severity) increases with the number of active substances in regular therapy (*R*^2^ = 0.264, *F*(1, 98) = 35.11, *p* < 0.001) (Figure 1). No statistically significant relationship was found between age and the total number of potential DDIs (*R*^2^ = 0.026, *F*(1, 98) = 2.61, *p* = 0.109).

After clinical evaluation of each interaction, 39 (27.5%) patients had at least one clinically meaningful DDI identified, including 38 (26.8%) patients having interactions of major significance and one (0.7%) having a potential contraindication DDI.

In the univariable logistic regression between the dependent variable "clinically significant potential DDI" and the different clinical and demographic variables from our patients (Table 3), age (odds ratio (OR) 0.94, *p* = 0.004), female sex (OR 4.93, *p* < 0.001), the number of active comorbidities (OR 0.51, *p* = 0.001), the Charlson Score (OR 0.70, *p* = 0.004), and the presence of proton pump inhibitors (PPIs) in regular therapy (OR 2.62, *p* = 0.031) were predictors of potential meaningful DDI. After adjustment through multivariable logistic regression (Table 4), only the female sex (OR 3.01, *p* = 0.029), the number of active comorbidities (OR 0.60, *p* = 0.029), and the presence of PPIs (OR 2.99, *p* = 0.033) remained as predictors of potential meaningful DDI.

**Table 3:** Univariable logistic regression (dependent variable "presence of clinically significant potential DDI"). 95% CI: 95% confidence interval; ECOG: Eastern Cooperative Oncology Group; DDI: drug-drug interaction; OR: odds ratio; PPIs: proton pump inhibitors.

Variables		95% CI		
	OR	Lower	Upper	p
Age	0.94	0.903	0.981	0.004
Female sex	4.93	2.063	11.772	<0.001
Metastatic disease	0.99	0.997	1.002	0.580
ECOG Performance Status	0.95	0.464	1.937	0.884
Number of active comorbidities	0.51	0.346	0.749	0.001
Charlson Comorbidity Index Score	0.70	0.546	0.894	0.004
Number of active substances in chronic medication	0.97	0.873	1.080	0.587
PPIs in chronic medication	2.62	1.092	6.311	0.031
≥5 active substances	1.15	0.387	3.416	0.801
≥10 active substances	0.61	0.243	1.512	0.283

**Table 4:** Multivariable logistic regression (dependent variable "presence of clinically significant potential DDI"). 95% CI: 95% confidence interval; DDI: drug-drug interaction; OR: odds ratio; PPIs: proton pump inhibitors.

Variables		95% CI		
	OR	Lower	Upper	p
Age	0.99	0.935	1.047	0.717
Female sex	3.01	1.116	8.093	0.029
Number of active comorbidities	0.60	0.385	0.949	0.029
Charlson Comorbidity Index Score	0.93	0.663	1.306	0.677
PPIs in chronic medication	2.99	1.093	8.200	0.033

## Discussion

The present study validated a drug interaction consulting service as a tool to support clinical decision-making, intending to reduce toxicity and/or minimize the impact on the effectiveness of oral anticancer treatments. It is worth noting that the frequency of patients with clinically significant DDI found (27.5%) exceeded the value established in the study's sample calculation (10%), highlighting the importance of this review and the availability of this service.

The main mechanisms of interaction found were induction or inhibition of metabolism, as well as potential conditioning of pharmacokinetics by interference with absorption. We also found pharmacodynamic interactions, such as the combination of QTc-prolonging drugs and hyperglycemia-associated agents, that could diminish the therapeutic effect of antidiabetic drugs.

Regarding potential DDI due to interference with absorption, we would like to highlight that 59.2% of patients had a long-term PPI in their usual therapy without always having a clear medical indication for its use. The DDI reports sent to the attending medical oncologist always included a suggestion for PPI indication review whenever they were part of the patient's chronic medication. PPIs have been found to have a potential interaction with CDK4/6 inhibitors and may reduce their effectiveness, particularly in the absorption of palbociclib. Some studies have shown that PPI use may be associated with decreased progression-free survival and overall survival in breast cancer patients treated with CDK4/6 inhibitors [24-27]. However, it is important to note that these findings are based on retrospective studies, which can only show an association and not a causal relationship. 

After statistical adjustment, our analysis found no evidence to suggest that age and the number of active substances were predictors of clinically meaningful DDI (major severity or contraindication) in our population. This suggests that the risk of such interactions is not limited to the elderly or those taking multiple medications. 

As stated before, DDIs are complex, and a single database search may not capture all potential interactions [20]. As expected, we found a significant rate of discordance between DDI databases for the same anticancer drug. Possible explanations are differences in the data sources, variations in the methods used to evaluate DDI, or the fact that new information about DDI is continually under investigation. Additionally, some databases may have more limited information on certain drugs or drug classes, such as newer or less commonly used anticancer drugs (e.g., tucatinib was not available in the RadBoudUMC Database at the time of this study). There were also variations in the level of detail provided for a specific interaction or in the interpretation of the available data.

This work has several limitations. First, patients had to be referred by their attending medical oncologist to participate in this project. This means that the oncologists may only have referred patients whenever they were in doubt of potential DDIs, implying that some patients at risk never had an opportunity for medication reconciliation. Secondly, we based our sample size estimation solely on the assumption that 10% of cancer patients would experience clinically meaningful DDI. However, we did not conduct sample size estimations for the linear and logistic regression analyses, which may have resulted in an underpowered analysis.

We would like to emphasize that the process of medication reconciliation and DDI search in different databases is time-consuming. As we recognize, polypharmacy should be an integral part of comprehensive cancer patient assessment and management. However, due to a shortage of time, medical oncology consultation may not be the correct place to perform this kind of work. 

In the future, the fast-growing application of artificial intelligence in healthcare may assist the process of medication reconciliation and DDI search with the cross-information of electronic prescriptions between different providers, as long as patients allow this kind of service due to the general data protection regulation.

## Conclusions

Although drug interactions are a concern in oncology, a systematic DDI review is rarely conducted in medical oncology consultations. The availability of a medication reconciliation service, carried out by a multidisciplinary team with dedicated time for this task, is an added value for safety enhancement in cancer patients. The fast-growing application of artificial intelligence in healthcare may facilitate this process in the future.
